# Multivocal Didaktik Modelling in Early Childhood Education—For a Sustainable Future in a World of Change

**DOI:** 10.3390/children10081419

**Published:** 2023-08-21

**Authors:** Ann-Christine Vallberg Roth, Linda Palla

**Affiliations:** Faculty of Education and Society, Malmö University, P.O. Box 50500, 202 50 Malmö, Sweden; linda.palla@mau.se

**Keywords:** abductive analysis, collaborative research, early childhood education, multivocal didaktik modelling, Swedish preschool, teaching realities

## Abstract

In times of democratic decline, unanimous choices and approaches and the idea of a singular best practice may be less conducive to democracy and sustainability. Therefore, the aim of this study is to suggest a multivocal approach to education and teaching by studying the question of what may characterise teaching in preschool for a sustainable future. The knowledge contribution and originality of the article is evident in the introduction, method, and results. In abductive analyses, models can summarize what we need to know and teach in pursuit of the creation of open life chances for every child. The results show that didaktik models are open and can provide support for teachers and leaders to consider and base informed decisions on, as well as to motivate their didaktik choices based on scientific foundations and proven experiences. Multivocal didaktik mo-delling intends to open up teaching—cultivating collaboration in preschool for a sustainable future in a world of change. In conclusion, we recommend cultivating an orientation (1) between knowledges, values and didaktik/education/special education in the pursuit of the creation of conditions for good education for all children; (2) between teaching realities and scientific foundations, which are founded in a critical–reflective didaktik, with a choice of direction in relation to an uncertain future; and (3) between continuity, progression and teaching adventures—which can include consolidating, deepening, broadening, raising, and cultivating knowledge and values for multivocality, democracy and sustainability in teaching realities. In the future the concept of multivocal didaktik modelling can be studied in relation to complex teaching realities as in a teaching universe or a teaching multiverse.

## 1. Introduction

This article strives to shed light on the concepts of multivocal and didaktik modelling based on analysis of collaborative research in two R&D programs and an Education, Learning, Research network (ELR, in Swedish: Utbildning, Lärande, Forskning (ULF) (see Funding Section) as a sustainable and democratic approach and tool for quality education for all children. In this conceptualizing approach, empirical elements are used as illustrative examples for the theoretical reasoning. In congruence with Goal 4.2 of Agenda 2030 [1], we strive to offer a potential preschool teaching solution to the challenges of social and environmental instability, fragility and uncertainty that Agenda 2030 is concerned with.

In this time of democratic decline in the Nordic countries as well as traces of global democratic regression [2], exclusively one-sided and unanimous choices—or “one right way and approach”—may be less conducive to democracy and sustainability than multivocal choices [3]. The term multivocal refers to multiple voices in many parts, including those of children, guardians, preschool teachers, work teams, school leaders, researchers, the state (e.g., curriculum and the Swedish Education Act [4]), global actors (e.g., United Nations, Agenda 2030) and the assemblies of the more-than-human [5]. The Norwegian linguist Dysthe [3] was inspired by the Russian literary theorist Bakhtin to launch “the multivocal classroom”. According to Liberg [6], ‘the multivocal word constituted a strong reaction against the authoritarian and unanimous—monological—Russian society in which they lived’ (p. 2).

With regard to the global Goal 4, on quality education for all, approximately 85 percent of all 1–5-year-old children and 95 percent of all 4–5-year-old children attend preschool in Sweden [1]. Having staff with relevant teacher training (pedagogic university degree) is crucial for ensuring quality in preschool education. While around 40% of preschool staff have relevant teacher training, this is lower than the 70% of teachers in elementary school who have adequate teacher training (ibid). In addition, the percentage of staff with teacher training and the size of children’s groups can vary in different municipalities (ibid). Furthermore, there can be a large difference regarding preschools’ operational quality and preschool teachers’ competence to teach [7]. Williams and Sheridan’s study shows that preschool quality varies, and suggests that ‘further research is needed to develop preschool teaching and didaktik, which benefits the group of children as well as individual children and contributes to an increase in the competence of preschool teachers’ ([7] p. 127). In other words, there are framework conditions to work with in the pursuit of equality and the achievement of global Goal 4 regarding quality education for all. Framework conditions such as the number of children in preschool, teacher competence and the size of children’s groups are fundamental for equality. However, this article is more process-oriented in relation to teaching in preschool. The article aims to contribute knowledge about what may characterise teaching in preschool based on a multivocal di-daktik perspective in the pursuit of a sustainable future in a world of change. In the article, a sustainable future is connected to Goal 4.2 of Agenda 2030. An overarching question thus guides the article: What can characterise teaching in preschool in the pursuit of a sustainable future in a world of change?

In addition to, and related with, issues of democracy, it can be said that we live in a social development characterised by fragility. The *Varieties of Democracy Report 2023* [8] reveals a concerning trend of global democratic regression, with advances made over the past 35 years being wiped out. Approximately 72% of the world’s population, or 5.7 billion people, now reside in autocracies, returning global democracy levels to those last seen in the 1980s. With reference to Sweden, Andersson et al. [9] assert:

The development of events within and outside the country’s borders makes clear how fragile much of what we take for granted can be. At the same time, it shows how humans themselves contribute to hurting both fellow humans and the earth where they live.

([9] p. 11)

In Sweden, basic freedoms and rights can mostly be seen as ‘for those who have the same views as yourself or those who are similar to the group you belong to’ ([10] p. 115) and important democratic principles have not the strong defence that we had expected, as follows:

More generally asked questions about support for democracy have in previous SOM surveys (Society, Opinion and Media Institute, University of Gothenburg) been interpreted as democracy having strong and undivided support among citizens in Sweden. When such a large percentage of Swedes claim that fundamental freedoms and rights are mostly for those who have the same views as oneself or those who are similar to the group one belongs to, it testifies that a deeper understanding of what democracy requires in the form of political tolerance is lacking in too many citizens.

([10] p. 115)

We argue that the aforementioned challenges are central to educators at all levels. The starting point in this article is therefore the idea that new societal conditions bring challenges for social cohesion, democracy and the well-being of individuals and opportunities for an equal and inclusive education. Today, education is emphasized as a central factor in counteracting segregation and exclusion. Early interventions for children’s development, learning and well-being correspond to an educational and research area that has received increased interest in recent years, locally, nationally and internationally from the respective perspectives of the EU, UNESCO and OECD. Increasing differences between children’s and students’ development, learning and well-being, and lack of achievement of goals in later school stages have affected engagements in preschool and the youngest children in many countries [11,12,13,14]. Lack of preparation for democracy places demands on research and development work throughout the school system. The present article can be seen as part of the context just described. By studying the question of what may characterise teaching in preschool for a sustainable future, multivocal di-daktik modelling can contribute to the pursuit of open life chances and well-being for every child, which in the long run can contribute to democracy in a wiser world.

A basic assumption in the article is that ‘there is actually no theory that can encompass the total teaching situation’ ([15] p. 131). Instead of proceeding from a “one size fits all” approach, we explore multivocality. This approach can contribute to the creation of conditions for the knowledge formation of all children, and accommodates and captures processes with knowledge and value dimensions, in complex teaching realities. In the article, we will formulate ourselves in terms of “we” to emphasize that multivocal didaktik modelling is advantageously examined in collaboration.

### 1.1. International, Nordic, and Swedish Research on Teaching and Didaktik in Preschool

In the Nordic tradition, Swedish preschool places itself with welfare state ambitions that are highly ranked in international reports [10,11]. Given that Nordic preschools have similar goal management systems, it is also easier to link, translate and use results from Nordic research than from non-Nordic studies in the present article. From a Nordic perspective, Sweden stands out in so far as preschool is a form of school that is regulated by a common education act for the entire school system [16], something which is not the case in the other Nordic countries. Teaching is mentioned in the acts and curricula of Finland and Iceland, but not in binding guidelines for daycare in Denmark and Barnehage in Norway (ibid). From an international perspective, longitudinal research points to the preschool teacher’s importance for the quality of the activities, and thus for children’s learning and development [17]. Furthermore, it appears that alternative forms of learning and teaching—such as interdisciplinary learning, e.g., theme-oriented/project-oriented; norm-critical learning, making alternatives visible in learning situations; and system-based learning, seeing the whole system with several levels and recognizing it as more than the sum of its parts—can be beneficial in relation to sustainable development [18]. UNESCO [14] has a focus on learning, child development and learning outcomes:

[…] from birth to five years should have a clear set of child development and learning outcomes that they work towards achieving in line with a broad child development, pedagogical and curriculum framework. Child caregivers and teachers should gain skills to work effectively with children from diverse backgrounds, needs and abilities in an inclusive manner. They should support and assess their progress in achieving expected outcomes and use assessment results to improve practice.

([14] p. 21)

The focus on children’s expected learning outcomes is also found in the Anglo-Saxon curriculum tradition.

#### 1.1.1. Features of Two Curricular Traditions

In international studies, different curriculum traditions can be viewed [19], such as the Nordic tradition and the “preschool as preparation to school” tradition [19]. Examples of these distinctive features can be described briefly and broadly, in a way that does not claim to account for different nuances and changing directions over time. The “preschool as preparation to school” tradition, also called the Anglo-Saxon tradition in OECD reports [20], is characterized by early childhood education being a preparation for school with more detailed and standardized goals for the children to achieve [18,19,20]. Schwandt [19] reports that ‘Children will be expected to reach pre-defined levels of learning goals to be achieved at each stage’ (p. 19). Prescribed target areas can relate to reading, writing and cognitive development. Assessment of standardized learning results—so-called “learning outcomes” at the individual level or “child outcomes”—is characteristic of the “preschool as preparation to school” tradition [11,19,20]. According to an OECD report [20], ‘Nordic countries tend to avoid using the term ‘child outcomes’, while Anglo-Saxon countries favour the approach’ (p. 1). Moreover, the Anglo-Saxon tradition can be interpreted as influenced by measurement theory and by goal-rational (goal- and result-oriented) thinking and management, which can relate to new public management (NPM) [21]. According to Biesta [22,23], the growing interest in measuring knowledge can be expressed in terms of a measurement culture. Measurement can be interpreted to hold claims to accuracy with higher confidence in certainty that can be expressed in numbers, while judgment/assessment instead refers to doing something that is not certain.

The Nordic tradition can be characterised by a more activity-oriented education, with goals to strive for in preschool activities, instead of goals to be achieved on an individual level, or knowledge requirements for the children of different ages. Documentation and pedagogical assessment are then not primarily used to make children’s development and learning visible at an individual level, but preferably to highlight the activities, the learning environment and the teachers’ working methods and input, even if in recent times there have also been elements of individual-oriented documentation and assessment [19]. In this context, teaching can be interpreted as being more compatible with the Nordic tradition than learning. Teaching is focused on creating conditions for learning, rather than focused on learning itself and on learning results based on knowledge requirements and achievement goals on an individual level.

#### 1.1.2. Teaching in Preschool and Didaktik Analysis in the Nordic Region

A research inventory on teaching in preschool in Sweden reveals a variety of theoretical approaches [13,24,25], including didaktik [26,27,28,29], developmental pedagogical [30], variation theory [31,32], pragmatic perspectives [33] and post-structural gateways [34,35,36,37]. The meaning of teaching in preschool [38] varies depending on which theoretical perspectives and inputs are used. When subject didaktik research at preschool is in focus [13], Swedish and Nordic studies emphasizing the “learning side” of didaktik. This has led to the identification of a so-called learnified didaktik [25]. In the present article, we connect to varying theoretical inputs (https://play.mau.se/media/t/0_zf8y4tp6, accessed on 16 August 2023) by showing empirical examples where participants in collaborative research attempt different theory-informed teaching arrangements linked to didaktik, variation theory, post-structural gateway and pragmatic perspective [39]. Overall, the theory-informed teaching arrangements are attempted and intertwined in the concept of multivocal di-daktik modelling.

A mapping of previous research with the keywords teaching, didactic/didaktik ana-lysis and preschool/early childhood education was conducted during the period 2010–2023 in scientific publications in the databases Ebsco, Eric and the Nordic Base of Early Childhood Education and Care (NB-ECEC). The mapping of Nordic and Swedish research shows that teaching-oriented didaktik analysis is a relatively young and undeveloped field, while learning-oriented analyses in preschool have a significantly stronger position in the research field [40]. In terms of method, researchers’ own analyses of qualitative data are more common than analyses of data in collaboration with professionals where scientific grounds meet proven experiences in the plural. By connecting to multivocality, we move from the expression of the singular ‘scientific ground and proven experience’ ([4] p. 5) to formulating ourselves in the plural ‘scientific grounds and proven experiences’. The lack of studies on teaching in preschool based on collaboration between researchers, preschool teachers and school leaders specifically focused on multivocal didaktik modelling motivates the present article.

### 1.2. Theory—Didaktik and Multivocal Didaktik Modelling in Relation to Complex Teaching Realities

**Figure 1:**
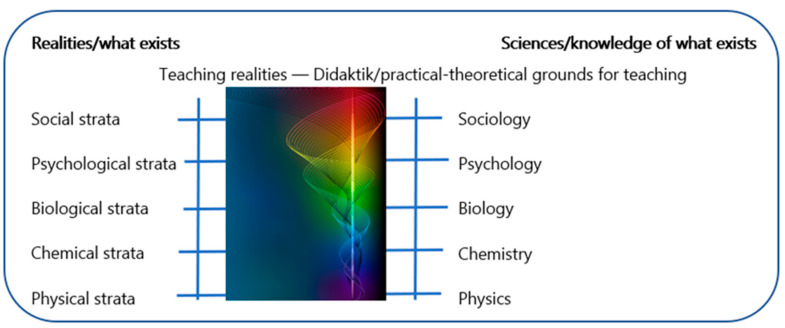
Areas of reality that exist in teaching realities and sciences/theories that can be linked to didaktik and illustrated in a meta-didaktik spiral (e.g., [39,43]; The authors have a license for the picture. The different colours can illustrate the multivocal testing of different theory-informed teaching arrangements. The meta-didaktik spiral is also presented as a virtual 3D-model in a film (2021): https://play.mau.se/media/t/0_zf8y4tp6, accessed on 16 August 2023.

The article connects to didaktik based on the aim and overall question. We could justify the necessity of didaktik by referring to governing documents—didaktik is prescribed, for example, in the Swedish Higher Education Ordinance (1993:100) [41]. Instead, the article takes the so-called reality, or rather realities, of preschool teaching as its starting point. The article refers to different areas of reality or strata, including physical, chemical, biological, psychological and social strata [42,43]. In teaching realities, all of these strata are contained and each of them is individually complex. Further, when research focuses on one of these strata, it is logical to start from the relevant field, such as physics for physical strata or sociology for social strata, and so on (see Figure 1). In relation to teaching, theories related to these respective strata become relevant as supporting theories. However, teaching cannot be reduced to these supporting theories. The viewing and acts will then be guided by theories in relation to areas of reality that are unable to capture the specialty and complexity within the teaching realities. For example, teaching is not just about existing in a physical or social reality. It is also an act of teaching about these areas of reality and creating conditions for actions and knowledge about these physical phenomena and social processes. In addition to teaching content such as plants and animals, physical phenomena, chemical processes, social relations and emotions, other content can emerge during the processes of teaching, such as mathematics, ethics, values, arts, history, language and interwoven, non-predetermined content. In these complex teaching realities, a multivocal approach can be attempted instead of a unilateral approach. This approach can be attempted not to reduce but instead to nuance and to create conditions for knowledge formation and experience in and with the world, and about these complex realities.

Figure 1 illustrates the intertwining through the representation of a meta-didaktik spiral of teaching realities and sciences/theories with a focus on didaktik as a multivocal teaching science [39]. If we direct attention to the upper part of the spiral, we see how the threads in the spiral enter from two directions. The threads from one side can symbolize the teaching realities in the municipalities, and the threads from the other side can symbolize multivocal teaching science. The threads from both sides converge in didaktik as a practical–theoretical knowledge base and begin to twist together. In this combination, theory-informed teaching arrangements are attempted, and are called overall didaktik models (see also Section 2.1), and which can be illustrated through the funnel shape with different colours in the spiral.

Biesta points out that ‘the argument here is that, while other disciplines can study educational processes and practices from their own angles, they do not have the devices to capture the reality of education as an educational reality’ ([44] p. 189). Thus, there is a particular need for didaktik research and didaktik analysis that is focused on contributing knowledge about what appears in teaching realities. Didaktik studies and analyses can be advantageously combined with other theories, but not reduced to other theories (see the right side of Figure 1). In what can be called ‘the didaktik curriculum’, ‘world knowledge’ becomes ‘teaching knowledge’ ([45] p. 76). It is then relevant that teaching can lead to both ‘discovery of the world and to participation in the world’ (ibid). Overall, this is about how world knowledge can be created in teaching and how knowledge can be created in the world. Thus far, we have justified why didaktik studies and analyses are needed. However, there are different didaktik orientations, and the question is which didaktik is meant in this article.

#### 1.2.1. Which Didaktik?

Didaktik is not a temporary trend and has been present since antiquity. Didaktik eras from antiquity to pre-modern, modern and late modern times have been described by didaktik professor Selander [46]. Didaktik gained wider impact through the writing of ‘Didactica Magna’—The Great Learning Doctrine—by Johan Amos Comenius in the seventeenth century. Comenius also brought forward his thoughts on teaching in the homes for the very youngest, 1–7-year-olds, in his ‘Informatium materum’—‘Mother’s School’, printed in 1632. Thus, there are historical traces of teaching and didaktik for the youngest children. Didaktik can be linked to both an Anglo-Saxon tradition (didactics, method-oriented and focused on ‘learning outcome’) and a continental/German/Nordic tradition [28,44]. This article links to the continental/German/Nordic tradition, which is indicated by the way that didaktik is written with the letter k instead of c.

Didaktik, or the Greek didaskein, means to teach, to point out and to direct someone’s attention to something. It can be about creating attention and adding something new to the co-action (action between at least two actors). Further, it concerns how world knowledge can be created in teaching and how knowledge can be created in the world. It refers to a didaktik where teaching is seen as a practical–theoretical interweaving that can accommodate the world of life and realities, and can also include the unknown and the more-than-human [47]. Teaching is a bundle of relational processes that can be created when, for example, teachers and children respond to each other so that a content can be created and interpreted together. Something simply emerges in co-action and in becoming: something is displayed [39].

Furthermore, didaktik can be seen, on the one hand, as a ‘scientific field that has a focus on teaching, but which also includes other scientific fields such as philosophy, history of ideas, psychology, sociology and political science.’ […]. On the other hand, general didaktik is also practical knowledge, a readiness for action, which is based on proven experience’ […] ([48] p. 57). Thus didaktik has both a practical and a theoretical side. The practical side focuses on experiences and proven experiences, while the theoretical side focuses on scientific grounds. In other words, didaktik constitutes a practical–theoretical knowledge base for teaching.

Examples of didaktik analysis can be linked to Klafki’s [49] content analysis, which is based on questions that are focused on the choice of content in teaching (with the di-daktik what-question at the centre). This analysis can be interpreted as focusing more on the content of planned and predetermined teaching than on spontaneous and non-predetermined teaching. In our critical–reflective didaktik, we expand and approach teaching based partially on both spontaneous and planned teaching, partially on several didaktik questions and partially on non-anthropocentric foundations with the material as an actor in non-predictable content that emerges in the teaching. That is, the more-than-human realities [5], of which the content is a part, are recognized in the didaktik analysis [47].

Wickman, Hamza, and Lundegård [50] emphasize the importance of didaktik analysis in relation to both established and new didaktik models for teachers and didaktik researchers. Didaktik analysis can be used to design and test new didaktik models, as well as to analyse established models. While didaktik analysis can be carried out without testing didaktik models, this article focuses on the use of didaktik models in analysing both planned and spontaneous teaching in preschool.

Planned teaching can be seen as co-planned based on didaktik issues [51]. Co-planned means that there are at least two actors, for example preschool teachers and work teams, or preschool teachers and children who plan the teaching together. Spontaneous teaching occurs in the moment. Jonsson [52] has coined the concept of didaktik of the present, which is focused on children’s play, learning and interests in the present and in which children’s experiences are taken care of and are linked to didaktik issues: ‘The teachers’ statements give an image that the activity is largely guided by the children’s demonstrated interests, needs and experiences and is about learning tangible, current content in a concrete, situationally appropriate way’ ([52] p. 104). Lind [53] maintains that ‘goal-relational work is less about making plans […]. It is more about making visible what can happen in a ‘planless’… work and thinking with the certainty that ‘anything’ can still happen or ‘become’ ([53] p. 94). With the revision of the Swedish Education Act ([4], Section 3, entered into force 2 January 2023) the definition of teaching has changed from “goal-directed processes” to “processes”. This change in definition can be interpreted as making it easier to include spontaneous and goal-relational teaching in the definition.

Some researchers emphasize that didaktik is the professional science of teaching as it revolves around teachers’ core activity of teaching [54,55]. In this context, didaktik mo-delling refers to the experimental shaping and reshaping of teaching and the analysis of teaching through cooperation between teachers, leaders and researchers. Our article touches on several didaktik models, but we focus on, test and exemplify, in particular, one didaktik model in the form of an alternative didaktik triangle (see Section 2.4). In the next section we deal with the question of what basis there can be for models—in other words, what scientific theoretical basis for models is meant in the article.

#### 1.2.2. Model Dependence and Conscious Realism—Hidden Reality

Didaktik and didaktik models can be linked to a variety of scientific theoretical foundations, including realism [46]. Realism has several meanings, which can refer to reality existing regardless of whether we see it (trees can fall in the forest even when we do not see them), or to the assumption that reality is preferably as it appears through our senses. According to what might be called model-dependent and conscious realism, it is ‘pointless to ask whether a model is real; the only meaningful thing is to ask whether it is consistent with the observations made’ ([56] p. 92) and the questions asked [46,57]. If two models both agree with the observations, then one cannot be said to be more real than the other. According to Hoffman, ‘there is an objective reality. But that reality is fundamentally different from our perceptions of objects in time and space’ ([56] p. 111). Our minds are limited in their ability to register everything in the reality, but instead register a hidden reality. For example, some animals have significantly better senses of smell, vision and hearing than we do. Based on the theory of evolution, it was precisely our human senses, which register a hidden reality, that turned out to be adapted for our good and our survival, which can then be expressed in terms of a conscious realism [56]. For example, if we had to register every atom before deciding what to eat, we would starve before we could taste our dinner.

Similar to that just mentioned, models can be seen as representations of hidden realities. Models do not represent reality in all of its complexity, but a partially hidden part of reality [56]. Certain aspects of the world are placed in focus by models. The point is whether the model is consistent with the observations made and the questions asked. Models can be seen as supports to guide us without describing a totally truthful reality in all respects and details or as supports that enable us to perform actions that benefit life in the world and our survival [56]. As with the example of food, teachers cannot at every moment act based on the complex teaching situation in all its physical, chemical, biological, psychological and social details and realms of reality. In their teaching realities, teachers cannot, for example, register what is happening in children’s brains and bodies and see if and when learning takes place. In that sense, learning belongs to a hidden reality. What we can do as teachers is to assess signs of learning in what is shown in actions and events. In that sense, teachers act based on a hidden reality and, within this, didaktik models can represent a reality that is adapted to co-actions in teaching situations. In didaktik models, the more-than-human reality, of which the content is a part, can also be represented. In summary, based on a model-dependent and conscious realism, there is a world that exists even when we do not see it [56]. However, our minds do not show that world—they do not reveal the so-called ‘true nature’ of reality. Our sense perceptions instead provide support for us to act in an adequate way, ‘admittedly in an incomplete but still functional way’ ([56] p. 159). Models can summarize what we need to know to live and to teach in the pursuit of creating open life chances for every child—open chances for more-than-human life—and well-being in every present moment [58]. Didaktik models in our article are not intended to provide precise and specific instructions for how teaching should be carried out in each individual case. Rather, they should be viewed as possible and open models that may provide support for teachers and leaders to consider and motivate their didaktik choices. These models are based on both scientific foundations and proven experiences.

#### 1.2.3. Multivocal Didaktik—Multivocal Didaktik Modelling

Adding to the reasoning behind the continental and Anglo-Saxon curriculum tradition, the continental tradition can be linked to general didaktik, while the Anglo-Saxon tradition can be linked to subject didaktik [44]. In this context, a dichotomy appears between general didaktik and subject didaktik [48]. It is urgent and relevant in relation to complex teaching realities in preschool to bridge the dichotomy between general didaktik and subject didaktik as these can be seen to a great extent as beneficially intertwined within the framework of preschool. The concept of “multivocal didaktik” and “multivocal didaktik modelling” can be attempted as an open didaktik and modelling that accommodates different variants and orientations, including general didaktik, content- and subject-oriented didaktik, and special education oriented didaktik [59]. Didaktik for a sustainable future in a world of change can be based on several didaktik voices that can be involved and tested.

#### 1.2.4. Examples of Didaktik Models

**Figure 2:**
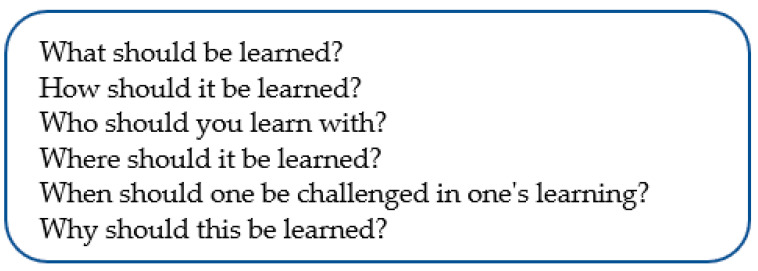
Classic verbal didaktik model with didaktik questions.

Classic didaktik models are the didaktik triangle [60,61] and didaktik questions. Di-daktik questions can be used both in practice and in analysis. In a classic verbal didaktik model, the didaktik questions are built into a traditionally prescriptive didaktik, with an emphasis on “should”—writings that are oriented towards learning [60], according to Figure 2.

The didactic model—which is focused on “what should be learned” and more (Figure 2) can partly be linked to a so-called learnified didaktik [25] and partly to an Anglo-Saxon “learning-outcome tradition” with a focus on what should be learned based on requirements for an individual level [19,62], and partly to learning as a hidden reality.

**Figure 3:**
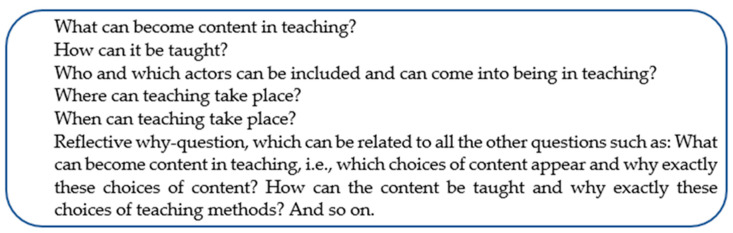
Alternative didaktik model—didaktik questions with “can” focused on teaching.

Instead, we attempted an alternative didaktik model where we replaced the modal auxiliary verb “should” with “can”. In this way, the model was placed in a critical–reflective didaktik that allows for teachers to make choices but recognizes that there are always alternatives to those choices. This approach emphasizes the importance of reflecting on choices within the framework of democracy. The model can be suitable for managing goals in the preschool setting. However, in the preschool, there are no “shall-goals” at the individual level in Nordic preschools. We also shifted the focus from learning to teaching in the revised model. The questions were formulated according to Figure 3.

Attempts to look beyond the here-and-now given can be interpreted as being in line with an unknown future and with didaktik questions that are formulated with “can” instead of “should” [51]. In other words, it regards an openness and curiosity about what could be and become. The didaktik questions in Figure 3 have been used by participants in the collaborative research teaching approach. Although the questions have also been used in collaborative research analyses, they are not at the forefront of this article.

In the context of the term multivocal didaktik, we can also find support in other di daktik models, such as didaktik levels [25,39,59,63]. Didaktik levels can be attributed to action level, theoretical level and meta-theoretical level [63]. In collaborative research, the action level can refer to traces of co-planning, conducting and co-evaluation of teaching with connection to a critical reflection of governing documents. The theoretical level can refer to traces when the participants scientifically ground their teaching and relate it to theoretical inputs and concepts. In collaborative research, this happens, for example, through didaktik, variation theory, post-structural and pragmatically informed teaching approaches [39]. The metatheoretical level can refer to evidence of participants placing and orienting themselves on a metatheoretical level relating to ontology and epistemology [25,39]. In collaborative research, there are traces in the material and teaching arrangements that can move between anthropocentric and non-anthropocentric foundations, between social constructionism, phenomenology, post-constructionism and realism (ibid). These are philosophical and theoretical foundations that have been touched by participants [25]. Furthermore, anchoring didaktik on a meta-theoretical level can contribute to the prevention of the didaktik floating around in an unawareness of basic assumptions and in a belief that all choices result in the same possibilities. When the choices are based on both the action level and the (meta)theoretical level, the justifications can become more precise and fine-tuned, and tactfully adapted to different conditions. In complex teaching realities, a multivocal approach can be attempted rather than a unanimous approach. This approach aims not to reduce but to nuance and create conditions for knowledge formation in the complex realities (see Figure 1). In complex teaching realities the professional teachers need tools to help them navigate. The relevance of didaktik modelling for professionals is further described in the following section.

#### 1.2.5. The Relevance of Didaktik Modelling as a Compass for Professionals

Metaphorically, didaktik modelling can be seen as the interaction between “map, terrain and compass”. Scientific basis and a “theoretically informed side” of the didaktik can then be seen as the map (knowledge of), while proven experience and the practically informed side of the didaktik can be seen as the terrain (familiarity with/knowledge in action). The point of didaktik modelling can then be described as an interaction between map and terrain, where both can challenge each other to create an expanded, refined and calibrated compass (knowledge for). The compass can be a potential tool (see for example Figure 3 and Section 3.1.2) for professionals to navigate in their further development of teaching in preschool. The navigation can be undertaken against the background of contemporary social challenges characterised by reduced equality and weakened democracy.

## 2. Materials and Methods

### 2.1. Methods and Context

Based on the aim and the overarching question, this article draws on data generated as part of collaborative research [64,65] in two R&D programs and an Education, Learning, Research (ELR) network (in Swedish: Utbildning, Lärande, Forskning (ULF), see Funding Section). As researchers, we did not select which school authorities/municipalities should participate in the research. A total of 20 school authorities/municipalities in Sweden initiated and chose to participate, while the participating school authorities/leaders made the selection of approximately 200 preschools/departments. As it was the participating school authorities/leaders who made the selection of preschools/departments and participants in the collaborative research, the selection methods were not known to the researchers (see also Section 4 in this article and [64,65]).

A total of 20 of Sweden’s 290 municipalities are included in the collaborative research, located in 6 of Sweden’s 21 regions (Stockholm, Sörmland, Uppsala, Örebro, Jönköping and Skåne). The six regions are located in central and southern Sweden and have varying sociodemographic characteristics [66]. The approximately 200 preschools/departments are located in (A) large cities/districts and municipalities close to large cities, (B) larger cities or near larger cities, and (C) smaller cities/towns and rural municipalities [66] (see further information about the municipalities in the Funding Section, links). In Sweden, 46 percent of the population aged 25–64 has some form of post-secondary education. The level of education is significantly higher in large cities and municipalities close to large cities compared with smaller cities and rural municipalities. In metropolitan areas (large cities and municipalities close to large cities), just under four out of ten are highly educated, while the proportion is half as large in smaller cities and rural municipalities [67].

The design of the collaborative research consisted of the testing of theory-informed teaching arrangements in the programs and the network. The researchers provided information at the start of each program through lectures and reference material [68,69]. Thereafter, the participants tested the teaching arrangements without the researchers being present in the preschools. In other words, the theory-informed teaching arrangements were tested by participants without the presence of researchers in the practices. The teaching arrangements were studied based on the participants’ written plans (co-planning); their teaching, which could be filmed and/or documented in other ways (e.g., audio-recorded, column-documented); and the participants’ written follow-ups of the teaching (co-evaluation) (see material in Table 1).

### 2.2. Method

A qualitative method, or more accurately an approach that processes qualitative data, was employed based on the theory [71]. Theory-informed teaching arrangements refer to the practice of participants of the various teaching arrangements informed by different theories [39]. In the article, we refer to theory in the sense of thinking tools as support for preschool teachers’ and leaders’ choices [72]—‘a set of thinking tools’ ([73] p. 5). Theories can provide support for, on the one hand, expanding and transcending everyday experiences and, on the other hand, reducing complex practices. In the collaborative research, the theory-informed arrangements connect to theories that the participants took up in descriptions of what can characterise teaching in a questionnaire that was answered at the start of each of the programs [25]. Theories that the participants explicitly touched on included didaktik, variation theory, post-constructionism and post-structural gateways, and pragmatic and sociocultural perspectives [25,39]. Furthermore, the teaching arrangements included theories related to content, such as literacy; music and digital technology; mathematics and programming; natural sciences, including chemistry; and movement, health and sustainability (see the Funding section in this article and [39,43]). Consistent content in the programs and the ELR network that could be related to global Goal 4 included literacy(s) and sustainability. We found the concept of the “theory-informed” teaching arrangement to be more feasible than, for example, “theory-based” teaching methods. This is because, in relation to our basic assumption, we wanted to further develop knowledge about teaching that is not only guided and based in theory, but also where the experiences and judgments of professionals also formed an important foundation. The concept of theory-informed teaching can include both scientific bases challenging proven experience and proven experience challenging scientific bases [39]. We used the term teaching arrangement instead of, for example, teaching method because we were not only focused on method and the didaktik ‘how’ question but also included several didaktik questions (see Figure 3). The theory-informed teaching arrangements can be seen as overall didaktik models.

### 2.3. Materials

In this article, examples are taken from the material from collaborative research conducted in two R&D programs and an ELR network between the years 2016 and 2023. The material consists of word data and audio-visual data. The total material—consisting of the number of documents, words and film hours in the programs and the network—is presented in Table 1.

Overall, examples presented in the results are selected because they relate to the aim and overarching question of the article and are directed at making explicit and representing variation and distinctive traces in the material in the most illustrative, clear and least bulky way [74].

### 2.4. Didaktik and Abductive Analysis

In designing and testing didaktik models in the collaborative research, didaktik and abduction were combined [51]. Continental/German/Nordic didaktik guided a so-called critical didaktik [27,28,44,47,48,49,75,76]. We attempted critical–reflective didaktik [61] as a theoretical basis for practice in relation to an uncertain future. Didaktik could then be linked to teaching as a process for ‘the encounter with an unknown future’ ([48] p. 57). Abductive analysis alternates between theory and empirics [77,78,79,80], ‘where both are gradually reinterpreted in light of each other’ ([81] p. 57). Abduction comes from the Latin ab (away) + dūcō (to lead), which can stand for something that ‘leads away’ [82]. It can be about discovering something that goes beyond the known, something that can be sensed and that can open the door for further developed knowledge. Here, the exchange between scientific basis and proven experience can also be involved. The abductive analysis can be compatible with the critical–reflective didaktik in the sense that the abduction in its empirical–theoretical exchange, or exchange between scientific basis and proven experience, has its counterpart in the practical–theoretical basis of the didaktik, which can open the door for teaching realities that not only are what is ‘here-and-now-given’ […] ‘but also includes what potentially can be achieved—and which in the moment merely reflects a vague possibility’ ([77] p. 31).

Abductive analysis in collaborative research can be closely related to democracy and sustainability. This is because the collaborative research, which is based on abductive logic, has given room for involved participation in terms of question formulation, selection, generation of data, collaborative exploratory conversations and analysis discussions [83]. Several theoretically informed teaching arrangements have been tested. Multivocality and a variety of different options for education can be interpreted as more favourable for sustainability and for democracy than exclusively one-sided and unanimous choices. In collaborative research, multivocality has been tested within the framework of democracy [27,28], which through the abductive analysis has resulted in multivocal didaktik analysis. All participants in collaborative research can be seen as knowledge users and potential knowledge developers in the abductive analysis. Abduction means closely listening to nuances in the empirical material and testing angles that are multivocal on both the action level and the (meta)theoretical level, which can be seen as a democratically oriented approach [51].

In complex preschool and teaching realities, we can at the action and (meta)theoretical level try a multivocal rather than a unanimous approach, in order not to reduce but instead to nuance and create conditions for contributing knowledge about the complex realities. This approach can lead to more complex, fine-tuned and adapted teaching, which in turn can contribute to a wiser world. In other words, the logic of abduction can add a critical, nuanced and openly change-oriented dimension to didaktik collaboration research within the framework of democracy in a wiser world [51].

**Figure 4:**
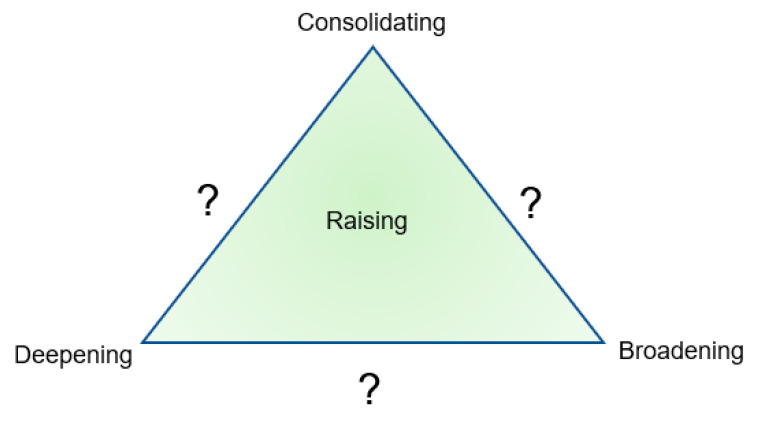
Multivocal didaktik model in the sense of creating conditions for relationships and movement between consolidating, deepening, broadening, raising, and cultivating knowledge and values that include the unpredictable, which is represented by question marks.

Overall, the didaktik analysis refers to teaching-cultivating collaboration and to designing didaktik models. In the article, we relate partly to established models and partly to testing and designing a specific didaktik model (see Figure 4). The article touches on didaktik questions (what, how, who/which, where, when and why), didaktik triangle (triangular relationship between teachers/leaders, children and content), didaktik phases (co-planning, conducting, and co-evaluation of teaching arrangements) and didaktik levels (action level, theoretical and meta-theoretical level) [39,51]. The theory-informed teaching arrangements can be seen as overall didaktik models that include the models just mentioned.

In our article, the concept of multivocal didaktik modelling is tested. The concept can offer alternative tools and strategies for critically reflected teaching in relation to each child’s growth in a preschool for all. As mentioned at the beginning of the article, the term multivocal refers to several voices in many parts, which can then be translated into several angles and variation of approaches. Overall, multivocal didaktik modelling can be used as a tool for critical reflection, which can include the perspectives (versions) of different actors, varying scientific bases and proven experiences. Inference using abduction can provide the conditions for broad participation in the research process. This is because an abductive logic is based on discoveries and creativity that can allow for new meanings—to see new contexts, to interpret meanings, to perceive new relationships. Abductive logic means that conclusions are not drawn in a single way but can be stimulated by a multivocal wealth of associations, different perspectives and versions of reality. As a transition to ethical considerations, abductive analysis can be emphasized as a substantiated choice from an ethical perspective based on collaboration, which includes the voices of several participants. Everyone in collaborative research can be seen as a knowledge bearer, knowledge user and potentially a knowledge developer in an abductive analysis.

### 2.5. Ethical Statements

Regarding the empirical processes this article relies on, the following can be noted. The study has taken into consideration ethical research guidelines [84,85] and has followed the Swedish Research Council’s [86,87] directives in all respects. Ethical considerations were made continuously throughout the process. Children/parents and participating preschool teachers, child minders, school principals and school leaders signed an informed consent form, and were informed that they have they have the right to suspend their participation at any time. The material was used for research purposes only and is stored securely at the university. A total of 16,768 individuals agreed to participate in the collaborative research (Undif, Fundif and the ELR network, see the Funding section), of which 13,562 are children/guardians and 3206 preschool teachers/childminders/principals, deputy principals/managers/other. An application for authorization was submitted to the Swedish Regional Ethical Review Committee in Lund (diary no. 2017–1014, approval date 10 January 2018), and the collaborative research commenced upon the response that no specific ethical trial was required.

## 3. Results—Examples of Multivocal Didaktik Modelling in Collaborative Research

In this conceptualizing article, empirical strands are used as illustrative examples for the theoretical reasoning that is aimed at calling in, clarifying and discussing the concepts of multivocality and didaktik modelling. The results relate to good education for all children ([1] Goal 4.2)—more specifically multivocal knowledge and value dimensions in teaching for all children—which is tested through didaktik modelling. This conceptualizing approach can be seen as an offer of a sustainable and democratic approach with the relevant tools in a time of instability, fragility and uncertainty.

### 3.1. Good Education for All Children—Multivocal Knowledge and Value Dimensions in Teaching

In the theory-informed teaching arrangements, there are traces of multivocal knowledge and value dimensions that can characterise good education for all children. Empirical traces are included here with an emphasis on consolidating, deepening, broadening, and raising knowledge and values (Section 3.1.1). Examples from empirical analysis are followed by a theoretical analysis step where the traces lead into continuity, progression and teaching adventures (Section 3.1.2). The results provide examples of abductive discovery of teaching adventures with unpredictable traces in the material (Section 3.1.3).

#### 3.1.1. Empirical Traces with an Emphasis on Consolidating, Deepening, Broadening, and Raising Knowledge and Values

Empirical traces are about consolidating, deepening, broadening, and raising knowledge and values. Although these dimensions are included in every teaching arrangement, some dimensions are distinctive (see Table 2), as follows: (1) in the didaktik arrangement, the dimension of consolidating knowledge and values is distinctive (see column 1.1 in Table 2); (2) in the variation theory and didaktik informed arrangement, the dimension of deepening knowledge and values is distinctive (see column 2.2 in Table 2); (3) in the post-structural and didaktik informed arrangement, the dimension of broadening knowledge and values is distinctive (see column 3.3 in Table 2); and (4) in the pragmatic and didaktik informed arrangement, the dimension of raising to global values is distinctive (see column 4.4 in Table 2).

#### 3.1.2. Continuity, Progression and Teaching Adventure—Theoretical Analysis including Testing of a Didaktik Model

Multivocal teaching can be interpreted as moving between continuity and progression [39,88]. Even Comenius’s [89] teaching principles can be interpreted as moving between continuity and progression. During the seventeenth century, Comenius was focused on a presentation of the art of teaching everyone everything [90]. Some examples of his principles are the following: the teaching should start with the simple and go to the complex—from the easy to the difficult, and from the known to the unknown. Teaching should proceed slowly, plan-wise and systematically without making any jumps. It must connect to the old ([89] p. 9). According to the preschool curriculum in Sweden 2018, the education must:

[…] make use of the children’s own experiences, needs and what they show interest in. But the children must also be continuously challenged further based on the curriculum by being inspired to new discoveries and knowledge. The preschool must contribute to continuity and progression in the children’s development and learning and prepare for continued education.

([91] p. 10)

Continuity in teaching can involve starting from the known contexts and starting with what children already know, can do and have experience of and show interest in [92]. Progression in teaching can refer to the creation of new contexts and building on knowledge, and creating conditions for growing, creating, progressing and deepening. Furthermore, it can also open opportunities to fantasize and co-create a previously unknown teaching adventure (ibid). Teachers then improvise in co-action with the children and the children’s wonder and questions. This can lead to open, unpredictable and unexpected teaching, which can be represented by the question marks in Figure 4.

Continuity aims at consolidating knowledge, while progression aims at deepening, broadening, and raising knowledge and values [92]. For example, consolidated knowledge can regard repeating, rehearsing, training, processing, acting, trying, revisiting, retelling, and teaching others (see quotation examples in columns 1,1, 2.1, 3.1 and 4.1 in Table 2). Repeating can be attributed to a didaktik point. However, “in repetition”, the same thing is not “just” repeated ad infinitum—something new happens with the known. Otherwise, ‘it would be difficult to understand how changes can take place through persistent repetition […]. What returns is always the difference in the similarity. Paradoxically expressed: in the repetition, something different is repeated, even if it is the same’ ([45] p. 96).

Deepening knowledge (see examples of quotations in columns 1.2, 2.2, 3.2 and 4.2 in Table 2) can mean, for example, that we create the conditions for paying attention, distinguishing, experiencing and exploring details and that we are focused on limited learning objects in teaching [93]. Broadening knowledge (see citation examples in columns 1.3, 2.3, 3.3 and 4.3 in Table 2) can mean making available more and new angles on a phenomenon than before. This can involve creating the conditions for new intertwined (transdisciplinary) combinations of content to emerge [94,95]. Raising can mean focusing on values and combining multiple dimensions of knowledge to a higher level (see quote examples in columns 1.4, 2.4, 3.4 and 4.4 in Table 2), that is, collaboratively approaching the complexities of reality and linking it to higher values such as global sustainability [33]. Overall, cultivating knowledge and values in unique teaching adventures can involve the multivocal dimensions of consolidating, deepening, broadening, and raising knowledge and values. These dimensions can be linked to good education for all: for a sustainable future in a world of change (see Figure 4).

#### 3.1.3. Teaching Adventure with Unpredictable Traces—Abductive Discovery

Teaching adventure accommodates the cultivation of knowledge and values that include the unpredictability and situational uniqueness of spontaneous teaching: ‘Like artists, teachers must improvise and build on events that develop during the teaching process. Much in teaching is unpredictable and the results are often unexpected’ ([96] p. 326). It can regard moving between different “co-swirling” contexts and knowledge in motion (see spiral in Figure 1 and question mark in Figure 4). The art of teaching can refer to the teacher’s skill in setting knowledge processes in motion (see Figure 1). It is also about teaching realities containing practical experiences and horizons of non-knowledge in the moment [97]. The following are examples of traces of non-knowing and unpredictability that occur spontaneously in co-planning and co-evaluation of the theory-informed teaching arrangements:

Exciting because you don’t know in advance what it will be.

(Undif-material in [83] p. 24)

[…] We don’t know where we are going […] can take any road. Start from the children and listen to them. More children can come forward when we go the roundabout (kringelikrokig) way.

(Fundif-material in [39] pp. 42–43)

Don’t know which way […] everything is possible…where it ends, we don’t know.

(ELR-material, 21 April 2023)

The above quotes show traces of not knowing in advance and that ‘everything is possible’. Therefore, multivocal teaching adventures can involve co-acting, co-creating and developing knowledge in (un)expected paths and crossing established boundaries, and navigating in (un)known waters and (un)equivalent worlds. Multivocality can also be seen as an opening of the possibility for something new, something that can sensitively open up to the uniqueness that can only be sensed at the moment. Furthermore, children’s right to a pluralistic education can be cited [98].

Everything in Figure 4 can be summarized as creating the conditions for multivocal dimensions of knowledge and values, which can then move between continuity, progression, and teaching adventure—in other words multivocal teaching and knowledge formation. Everything in Figure 1, Figure 3 and Figure 4 and Table 1 and Table 2 is tested in the unifying concept of “multivocal didaktik modelling”, with multivocality at the action level and (meta)theoretical level.

## 4. Discussion—Multivocal Didaktik Modelling for a Sustainable Future in a World of Change

The article aims to contribute knowledge about what can characterise teaching in preschool based on a multivocal didaktik approach in the pursuit of a sustainable future in a world of change. The overarching question was: What can characterise teaching in preschool in the pursuit of a sustainable future in a world of change? Multivocality [3] and a variety of different options for teaching can be interpreted as more favourable to sustainability and democracy than unanimous and unilateral choices. In this article, the multivocal has been extended to both action and (meta)theoretical levels, in comparison with Dysthe [3], who was preferably multivocal at the action level in “the multivocal classroom”. According to extensive studies, a wave of democratic decline, so-called autocratisation, is taking place in the world, where more countries are becoming more authorita-rian and less democratic [2]. Considering this, it becomes urgent in both national and international perspectives to protect freedom and democracy and share multivocal examples: ‘In a democracy, it must always be a diversity of values that are important, a diversity that is also constantly renegotiated’ ([97] p. 143).

Theory-informed teaching arrangements have been tested and shown to be feasible as overall didaktik models in the two R&D programs and in the ELR network. They have enabled teaching development and varied didaktik modelling in relation to partly different values and knowledge, and partly spontaneous and planned teaching in relation to groups of children and unique children. Teaching has resulted in multivocal traces at the action level and theoretical levels [39].

Abductive analysis in the article is a collaborative approach that seeks to explore the unknown and the possible, arousing curiosity and bridging gaps between teaching realities and knowledge. In educational contexts, collaboration and shared understanding are essential, and abductive research facilitates this by allowing practitioners and researchers to collaborate on complex and composite issues. By interweaving different perspectives and gradually trying and developing useful didaktik models, abductive research contributes to a possible direction in exploring what is educationally feasible. The abductive process is a long and iterative one, generating insights that can help bridge the gap between empiricism and theory, resulting in both fruitful and credible models [79,80].

Multivocal didaktik modelling can correspond to the need to try different orientations in the encounter with multifaceted teaching realities and multifaceted content and question areas: ‘one is served by a rich set of both methods and theories. By being oriented in what is available, one can make use of what is best suited for different issues, orientations, and purposes’ ([99] p. 58). Good education for all children is concretized and tested in the article through multivocal didaktik modelling with a focus on knowledge and value dimensions in teaching for all children.

### Method Reflection—Limitations, Reliability, and Usability

Our chosen collaboration method refers to collaboration with participants through the research part of the R&D programs and the ELR network—from the initiation of questions about teaching in preschool, to dialogue about research questions, to the choice of design for theoretically informed teaching arrangements, to the selection of preschools/departments and participants, to the generation of data and discussion of the abductive outcome of the analysis [39,51,64,92]. In this large-scale collaboration method, it was the participating school authorities/leaders who made the selection of preschools/departments and participants, and the researchers were not directly present in the preschool practices. Instead, the researchers only took part in practices that were registered by those working in the selected preschools from school authorities/municipalities that have chosen to participate in the collaborative research, which may have resulted in a “positive selection”. This means that there may be much that has happened in the preschools that has not been registered and that the researchers cannot comment on [39]. In that sense, the choice of method was consistent with the collaborative research’s aim and overall question, which was focused on what “can” characterise teaching in preschool. In other words, we do not claim to know what characterizes teaching in preschool. Instead, we aim to contribute knowledge about what can characterize teaching in preschool. This method design involved researchers not being directly present in preschool practices and only taking part in registered practices. The participants generated the data and chose what they wanted to show the researchers. This method design has proven to be feasible even in times when physical presence was reduced, such as during the Covid pandemic.

To ensure the confidentiality and security of recorded data, the researchers followed legal and ethical guidelines. The platform used to store the data met the legal and security requirements imposed on a Swedish authority. Additionally, one participant per preschool/department was appointed to post material on the platform, and the designated person only had access to the material they uploaded themselves. However, the researchers acknowledge that the participants chose which material would be posted on the platform, which may have resulted in a “positive selection” of documents. In other words, there may have been teaching situations that the researchers were not allowed to take part in and that deviated from what we analysed.

There are different ways of using and dealing with criticism. An example is to see criticism as a way to relatively one-sidedly emphasize negative meaning, such as flaws, inherent contradictions and shortcomings [100]. However, we try to cultivate a concept of criticism that is multivocal and “multi-turnable”, which allows us to turn, twist and look at both opportunities and challenges with our choices. We attribute this to critical–reflective didaktik. We are then not unilaterally focused on destabilizing and deconstructing but are also focused on possible reconstructions of models. Ferraris [101] emphasizes that ‘any deconstruction without reconstruction is irresponsible’ (p. 9).

The results can be understood in terms of situated generalization, which means that the results provide alternative perspectives and concepts rather than one truth [102]. With such an approach, the generalization of the results cannot be predicted, but takes place through recognition. Recognition happens when the reader can recognize identified traces of teaching and can use results and concepts as tools in their practice. By using this approach, the situated generalisation means that the results are open to further interpretation and offer guidance to readers in similar cases, situations and contexts outside of collaboration research.

Overall, the degree of reliability, plausibility and usability can be said to be highly based on the fact that the collaborative research was carried out in 20 Swedish municipalities/school authorities and around 200 preschools located in (A) large cities/districts and municipalities close to large cities, (B) larger cities and near to larger cities, and (C) smaller cities/towns and rural municipalities [66], during the years 2016 to 2023. Through the concept of multivocal didaktik modelling, preschool teachers and leaders can get more stable support and better conditions to act judiciously in relation to concrete teaching situations, which can lead to more composed, fine-tuned and adapted teaching in complex teaching realities. These results could be informative and useful for both university programs for ECE teachers and professional development courses and programs. The multivocal di-daktik models can be further tested in future studies, in teaching practices and in university programs for ECE.

## 5. Multivocal Didaktik Modelling—Conclusions and Future Research

In summary this is a conceptualizing article with empirical examples. The article highlights the concepts of multivocal and didaktik modelling based on analysis in colla-boration as a sustainable and democratic approach and as a tool that strives for quality education for all children in relation to Goal 4.2 of Agenda 2030. We have presented a complex theoretical framework and used examples to aid understanding. Section 1 in the article has sufficient background, including several relevant references, to focus on the Nordic/Swedish context and secondarily to the continental/German context rather than the dominant Anglo-Saxon one. This is seen as a strength, and an original aspect of the article in connection to Figure 1, Figure 3 and Figure 4. The study is based on a large-scale co-research design. The research design is appropriate to the aim and the overarching question of the article and has been tested for eight years, as described in Section 2. The results are provided as examples and the conclusions are supported by the presented results.

The original aspects and the knowledge contribution in this conceptualizing article with empirical examples emerge in relation to early research, firstly, in Section 1, we highlight multivocal didaktik modelling (see Figure 1 and Figure 3), graphically illustrated in a meta-didaktik spiral, as a sustainable approach and tool that strives for quality education for all children in relation to complex teaching realities in a time of democratic decline. Secondly, this paper contributed, in Section 2, via a large-scale co-research design with abductive analysis compatible with the continental critical–reflective didaktik, and in a design that had been tested for eight years in 20 school authorities/municipalities and approximately 200 preschools/departments in Sweden with a total of 16,768 individuals who had agreed to participate. This research design was also able to generate robust empirical material in the time of the Covid pandemic (see Table 1). Thirdly, this article contributes, in its Section 3, via a multivocal didaktik model (see Figure 4) that opens up for teaching in preschool between continuity, progression and teaching adventures—including consolidating, deepening, broadening, raising (see Table 2), and cultivating knowledge and values for multivocality, democracy and sustainability in complex teaching realities.

Multivocal didaktik modelling intends to open up teaching—cultivating collaboration in preschool for a sustainable future in a world of change. In summary, it can mean cultivating the following orientations;

1. between knowledges, values, and didaktik/education/special education in the pursuit of the creation of conditions for good education for all children;
2. between teaching realities and scientific foundations, which are founded in critical–reflective didaktik with a choice of direction but without a predetermined end goal in relation to an uncertain future;
3. between continuity, progression and teaching adventures—which can include consolidating, deepening, broadening, raising, and cultivating knowledge and values for multivocality, democracy and sustainability in complex teaching realities.

Finally, the overall point and key result is that multivocal didaktik modelling can rally support for the idea of making thoughtful choices in the effort to create conditions for the open life chances and well-being of every child in every present moment [58] and open up chances for more-than-human life [5]. Multivocal didaktik modelling offers a way to address the complexities of teaching realities. By incorporating multiple perspectives and multiple voices in many parts, this approach acknowledges the diverse and multifaceted realities of educational contexts. It allows for a richer understanding of the complexities involved in teaching and enables the creation of more comprehensive and nuanced didaktik models. By considering various viewpoints, multivocal didaktik modelling helps to capture the teaching realities and provides a more holistic and inclusive approach to educational practices. Overall, the collaborative research’s knowledge contribution can be described in terms of theory-informed practice development and practice-based concept and theory development [103]. The concept of multivocal didaktik modelling can be further tested in future studies—for a sustainable future in a world of change. In the future the concept of multivocal didaktik modelling can be studied in relation to complex teaching realities as in a teaching universe or a teaching multiverse.

## Figures and Tables

**Table 1:** Total material from collaborative research in two R&D programs and one ELR network.

Collaborative Research	Number of Documents *	Number of Words	Number of Film Hours
R&D program Undif **	ca 2260	ca 479,000	ca 110
R&D program Fundif ***	ca 1460	ca 300,000	ca 35
ELR network ****	ca 320	ca 64,400	ca 10
Total	ca 4040	ca 843,400	ca 155

* Documents include co-planning and co-evaluations of theory-informed teaching arrangements that are based on didaktik questions (see Figure 3). Co-planning and co-evaluation mean that there are at least two actors, for example preschool teachers and work teams, or preschool teachers and children who plan and evaluate the teaching together. ** The R&D program Teaching in Preschool, Undif https://ifous.se/undervisning-i-forskolan/, accessed on 16 August 2023. *** The R&D program Multivocal Teaching in Preschool, Fundif https://mau.se/en/research/projects/multivocal-didactic-modelling-in-preschool/, accessed on 16 August 2023. **** The ELR network Didaktik Modelling in Preschool. ELR stands for Education (Utbildning), Learning (Lärande) and Research (Forskning) [70].

**Table 2:** Empirical traces with an emphasis on consolidating, deepening, broadening, and raising knowledge and values in theory-informed teaching arrangements.

Theory-Informed Teaching Arrangement	Consolidating	Deepening	Broadening	Raising
1 Didaktik-informed teaching arrangement	1.1 Distinctive traces ‘to repeatedly name the concept’. ‘During a few teaching hours, the learning objective is repeated on several occasions.’ ‘that the children recount different events to each other.’ ‘The children are actors in that they are the ones who come up with suggestions and teach the fairy tale character’. ‘Since we had already listened to the songs before and talked about the concept of pulse, the children recognized the songs and quickly found the pulse. Repetition is good for children.’	1.2 ‘enabled the children to deepen their learning’. ‘deepen children’s concepts in music. Feel pace, pulse, fast, slow’	1.3 ‘At the same time provides a breadth within the subject.’	1.4 Raising equality—‘helicopter perspective’. ‘The sustainability concept was well-being’
2 Didaktik and variation theory-informed teaching arrangement	2.1 ‘We will name the words big and small to consolidate.’ ‘We also had the same teaching sessions several times as the younger children are in greater need than the older ones of repetition before we can move on.’	2.2 Distinctive traces ‘learning object, where we can go more in depth on a certain topic.’ ‘That’s why I wanted to go a little deeper both in why you can experience the feeling of anger and how it can be expressed.’ ‘Becomes in-depth learning for the children.’ ‘We want the children to gain a deeper understanding of patterns by pointing to patterns that exist in our surroundings’. ‘An opportunity to narrow down in an area of knowledge and go down in depth.’	2.3 ‘Broaden children’s understanding of trains and other work vehicles.’	2.4 ‘The aim is to challenge the children’s mathematical reasoning connected to measurement and also to relate this to an equality aspect’
3 Didaktik and post-structural informed teaching arrangement	3.1 ‘repetition needs younger children’. ‘it took a few repetitions’	3.2 ‘Interesting that it was possible to do in-depth studies in certain subjects’	3.3 Distinctive traces ‘to work with many different forms of expression and transdisciplinary. It enables us to weave in many curriculum goals within the same project and at the same time find the children’s voices in it. ‘ ‘Unlike a variation-theoretical approach with a narrow learning goal, here we have been able to choose a theme/project that was overall and comprehensive, where we then had different directions that can be interpreted as rhizomatic.’ ‘Instead of choosing only one trace and following its direction, we have been able to provide the opportunity for several different traces from the start. The different directions have also contributed to a trans-disciplinary way of working, where several subjects intertwine with each other.’ ‘Materials and environments have also been given a greater role than usual in our teaching.’	3.4 ‘To raise the value of children’s participation and influence in the different parts of the preschool’
4 Didaktik and pragmatic informed teaching arrangement	4.1 ‘Through action, it becomes easier for the children to consolidate their knowledge.’	4.2 ‘A possible entrance to deepening the teaching around children’s influence.’ ‘[...] when you go more in-depth with what works and what doesn’t work [...]’	4.3 ‘There are several threads to build on. We ended up in discussions about what creates good conditions for cooperation. What opportunity do we give the children to build relationships with everyone (the breadth)’	4.4 Distinctive traces ‘We could see that the global goal, which deals with reduced inequality, gained great impact based on the fact that the conflicts were reduced and ‘we could observe that the children gained increased motivation to influence everything from small to large’. ‘makes it possible to capture spontaneous learning opportunities where different values are in focus.’ ‘We want to make the children aware that there are global goals that the whole world must reach and strive for.’

## Data Availability

Not available due to ethics.

## References

[1] SCB, Statistiska Centralbyrån (2022). Agenda 2030: Mål 4 God Utbildning.

[2] Lührmann A., Maerz S.F., Grahn S., Alizada N., Gastaldi L., Hellmeier S., Hindle G., Lindberg S.I. (2020). Autocratization Surges—Resistance Grows: Democracy Report 2020.

[3] Dysthe O. (1993). Writing and Talking to Learn: A Theory-Based, Interpretive Study in Three Classrooms in the USA and Norway.

[4] SFS, Svensk Författningssamling (2010:800) (2010). Skollagen, The Education Act.

[5] Haraway D. (2016). Staying with the Trouble: Making Kin in the Chthulucene.

[6] Liberg C. (2003). Flerstämmighet, skolan och samhällsuppdraget, Multivocalism, the school and the social mission. Utbild. Och Demokrati. Tidskr. Didakt. Och Utbildningspolitik.

[7] Williams P., Sheridan S. (2018). Förskollärarkompetens—Skärningspunkt i undervisningens kvalitet, Preschool competence. Barn.

[8] Papada E., Altman D., Angiolillo F., Gastaldi L., Köhler T., Lundstedt M., Natsika N., Nord M., Sato Y., Wiebrecht F. (2023). Defiance in the Face of Autocratization. Democracy Report 2023.

[9] Andersson U., Oscarsson H., Rönnerstrand B., Theorin N. (2022). Du Sköra ny Värld: SOM-Undersökningen 2021 SOM-Rapport nr 81.

[10] Persson T., Widmalm S., Andersson U., Oscarsson H., Oscarsson B., Theorin N. (2022). Politisk tolerans och självcensur i orostider. Du Sköra ny Värld: SOM-Undersökningen 2021 SOM-Rapport nr 81.

[11] OECD (2006). Starting Strong II. Early Childhood Education and Care.

[12] OECD (2013). Literature Review on Monitoring Quality in Early Childhood Education and Care (ECEC).

[13] Tallberg Broman I. (2015). Förskola: Tidig Intervention—SKOLFORSK.

[14] UNESCO—United Nations Educational, Scientific and Cultural Organisation (2022). Global Partnership Strategy for Early Childhood, 2021–2030.

[15] Arfwedson G. (1998). Undervisningens Teorier och Praktiker: Didactica 6.

[16] Vallberg Roth A.-C. (2015). Nordisk komparativ analys av riktlinjer gällande innehåll och kvalitet i förskola, Nordic comparative analysis of guidelines regarding content and quality in preschool. CEPRA-STRIBEN Tidsskr. Eval. Praksis.

[17] Sylva K., Melhuish E., Sammons P., Siraj-Blatchford I., Taggart S. (2010). Early Childhood Matters: Evidence from the Effective Pre-School and Primary Education Project.

[18] UNESCO—United Nations Educational, Scientific and Cultural Organisation (2012). Shaping the Education of Tomorrow: The 2012 Report on the UN Decade of Education for Sustainable Development.

[19] Bennet J. (2010). Pedagogy in Early Childhood Services with Special reference to Nordic Approaches. Psychol. Sci. Educ..

[20] OECD (2012). Starting Strong III: A Quality Toolbox for Early Childhood Education and Care.

[21] Schwandt T.A. (2012). Quality, standards and accountability: An uneasy alliance. Educ. Inq..

[22] Biesta G. (2011). God Utbildning i Mätningens Tidevarv.

[23] Biesta G. (2017). The Rediscovery of Teaching.

[24] Sheridan S., Williams P. (2018). Undervisning i Förskolan: En Kunskapsöversikt.

[25] Vallberg Roth A.-C. (2020). What may characterise teaching in preschool? The written descriptions of Swedish preschool teachers and managers in 2016. Scand. J. Educ. Res..

[26] Bengtsson J. (1997). Didaktiska dimensioner: Möjligheter och gränser för en integrerad didaktik, Didactical dimensions. Pedagog. Forsk. Sver..

[27] Broström S. (2012). Curriculum in preschool: Adjustment or a possible liberation?. Nord. Early Child. Educ. Res..

[28] Broström S. (2022). Didaktik in preschool—Critical-democratic and play-oriented. Educ. Vetenskapliga Skr..

[29] Catucci E. (2018). Att Undervisa de Yngsta Barnen i Förskolan. Ph.D. Thesis.

[30] Jonsson A., Williams P., Samuelsson I. (2017). Undervisningsbegreppet och dess innebörder uttryckta av förskolans lärare, The teaching concept and its meaning expressed by the preschool teachers. Forsk. Om Undervis. Och Lärande.

[31] Thulin S., Gustavsson L. (2017). Lärares uppfattningar av undervisning och naturvetenskap som innehåll i förskolans verksamhet, Teachers’ perceptions of teaching and science as content in preschool activities. NorDiNa.

[32] Ljung-Djärf A., Holmqvist Olander M. (2013). Using Learning Study to Understand Preschoolers’ Learning: Challenges and Possibilities. Int. J. Early Child..

[33] Hedefalk M. (2014). Förskola för Hållbar Utveckling: Förutsättningar för Barns Utveckling av Handlingskompetens för Hållbar Utveckling, Preschool for Sustainable Development. Ph.D. Thesis.

[34] Lykke N. (2011). The Timeliness of Post-Constructionism. NORA Nord. J. Fem. Gend. Res..

[35] Nilsson M., Lecusay R., Alnervik K., Ferholt B. (2018). Iscensättning av undervisning: Målrelationellt lärande i förskolan, Staging of Teaching. Barn.

[36] Olsson L.M., Dahlberg G., Theorell E. (2016). Displacing identity—Placing aesthetics: Early childhood literacy in a globalized world. Discourse Stud. Cult. Politics Educ..

[37] Palmer A. (2010). Att bli Matematisk: Matematisk Subjektivitet Och Genus i Lärarutbildningen för de Yngre Åldrarna, Becoming Mathematical. Ph.D. Thesis.

[38] Rosenqvist M.M. (2000). Undervisning i förskolan? En Studie av Förskollärarstuderandes Föreställningar, Teaching in Preschool?. Ph.D. Thesis.

[39] Vallberg Roth A.-C., Aasa S., Ekberg J.-E., Holmberg Y., Sjöström J., Stensson C. (2021). Undervisning i Förskolan: Flerstämmig Didaktisk Modellering—Ifous 2021:4.

[40] Furenes M.I., Reikerås E., Moser T., Munthe E. (2021). Trender i Empirisk Barnehageforskning i de Skandinaviske Landene 2006–2019—En Forskningskartlegging Trends in Empirical Kindergarten Research in the Scandinavian Countries 2006–2019.

[41] (1993). Swedish Higher Education Ordinance (1993:100).

[42] Brandén H. (2015). Kritisk Realism.

[43] Vallberg Roth A.-C. (2022). Teaching in preschool: Multivocal didaktik modelling in a collaborative conceptual replication study. Educ. Vetenskapliga Skr..

[44] Biesta G. (2011). Disciplines and theory in the academic study of education: A comparative analysis of the Anglo-American and Continental construction of the field. Pedagog. Cult. Soc..

[45] von Oettingen A. (2018). Allmän Didaktik—Mellan Normativitet Och Evidens.

[46] Selander S. (2017). Didaktiken Efter Vygotskij: Design för Lärande.

[47] Bengtsson S. (2022). Didaktiken efter idealismen, Didactics after idealism. Pedagog. Forsk. Sver..

[48] Brante G. (2016). Allmän didaktik och ämnesdidaktik—En inledande diskussion kring gränser och anspråk, General didactics and subject didactics. Nord. Tidskr. Allmän Didakt..

[49] Klafki W. (1995). Didactic analysis as the core of preparation of instruction. J. Curric. Stud..

[50] Wickman P.-O., Hamza K., Lundegård I. (2018). Didaktik och didaktiska modeller för undervisning i naturvetenskapliga ämnen, Didactics and didactic models for teaching science subjects. NorDiNa.

[51] Vallberg Roth A.-C. (2022). Abduktiv analys i samverkansforskning—Fokus på didaktiska modeller i förskola, Abductive analysis in collaborative research. Pedagog. Forsk. Sver..

[52] Jonsson A. (2011). Nuets Didaktik—Förskolans Lärare Talar om Läroplan för de Yngsta, Didactic of the Present. Ph.D. Thesis.

[53] Lind U. (2010). Blickens Ordning: Bildspråk och Estetiska Lärprocesser som Kulturform och Kunskapsform, The Order of the Gaze. Ph.D. Thesis.

[54] Ingerman Å., Wickman P.-O., Burnard P., Apelgren B.-M., Cabaroglu N. (2015). Towards a teachers’ professional discipline. Transformative Teacher Research: In Theory and Practice for the C21st.

[55] Seel H., Hudson B., Buchberger F., Kansanen P., Seel H. (1999). Didaktik as the professional science of teachers. Didaktik/Fachdidaktik as the Science(-s) of the Teaching Profession?.

[56] Hoffman D. (2021). Dold Verklighet: Om Evolution, Medvetande och Perception.

[57] Selander S., Boistrup L., Selander S. (2021). Designs in and for learning—A theoretical framework. Designs for Research, Teaching and Learning: A Framework for Future Education.

[58] Trondman M., Tallberg Broman I. (2011). Snälla fröknar—Om barns perspektiv på barnperspektiv, Kind teachers. Skola och Barndom.

[59] Palla L., Vallberg Roth A.-C. (2020). Inclusive ideals and special educational tools in and out of tact: Didactical voices on teaching in language and communication in Swedish early childhood education. Int. J. Early Years Educ..

[60] Jank W., Meyer H. (2019). Didaktische Modelle, Dicatic Models.

[61] Uljens M. (1997). Didaktik—Teori, Reflektion och Praktik.

[62] OECD (2020). Early Learning and Child Well-Being: A Study of Five-Year-Olds in England, Estonia, and the United States.

[63] Kansanen P., Kansanen P. (1993). An outline for a model of teachers’ pedagogical thinking. Discussion on Some Educational Issues IV.

[64] (2018). Forska tillsamm ans—Samverkan för lärande och förbättring: Betänkande av utredningen om praktiknära skolforskning i samverkan, Research together—Collaboration for learning and improvement: Report of the inquiry into practice-based school research in collaboration.

[65] Nilsson L., Sorbring E. (2019). Samverkansforskning: Att Främja Barns och Ungas Välfärd, Collaborative Research: To Promote the Welfare of Children and Young People.

[66] Swedish Association of Local Authorities and Regions—SALAR (2023). Municipalities and Regions. https://skr.se/skr/englishpages/municipalitiesandregions.1088.html.

[67] SCB—Statistics Sweden (2022). Befolkningens Utbildning 2022, Educational Attainment of the Population. https://www.scb.se/UF0506.

[68] Vallberg Roth A.-C. (2016). Undervisning i Förskola: Flerstämmig Undervisning och Sambedömning i Förskola, Teaching in Preschool. Referensmaterial för Deltagare i FoU-Programmet.

[69] Vallberg Roth A.-C. (2018). Undervisning och Sambedömning i Förskola: Flerstämmig Didaktisk Modellering? Teaching and Co-Assessment in Preschool.

[70] Swedish Research Council (2023). Forskningsöversikt 2023—Utbildningsvetenskap.

[71] Åsberg R. (2001). Det finns inga kvalitativa metoder—Och inga kvantitativa heller för den delen: Det kvalitativa-kvantitativa argumentets missvisande retorik, There are no qualitative methods—And no quantitative either for that matter: About the misuse of the qualitative-quantitative argument. Pedagog. Forsk. Sver..

[72] Serder M., Jobér A. (2021). Vetenskapliga Teorier för Lärare.

[73] Wacquant L. (1989). Towards a reflexive sociology: A workshop with Pierre Bourdieu. Sociol. Theory.

[74] Derry S.J., Pea R.D., Barron B., Engle R.A., Erickson F., Goldman R., Hall R., Koschmann T., Lemke J.L., Sherin M.G. (2010). Conducting Video Research in the Learning Sciences: Guidance on Selection, Analysis, Technology, and Ethics. J. Learn. Sci..

[75] Biesta G. (2010). Why ‘What Works’ still won’t work: From evidence-based education to value-based education. Stud. Philos. Educ..

[76] Klafki W., Uljens M. (1997). Kritisk-konstruktiv didaktik. M. Didaktik—Teori, Reflektion och Praktik.

[77] Peirce C.S. (1903/1990). Pragmatism och Kosmologi: Valda Uppsatser i Översättning av Richard Matz och Med Inledning av Margareta Bertilsson och Peder Voetmann Christiansen.

[78] Tavory I., Timmermans S. (2014). Abductive Analysis: Theorizing Qualitative Research.

[79] Löfberg A. (2022). Abduktion som kunskapsfilosofi och forskningsmetod, Abduction as a philosophy of knowledge as well as a research method. Pedagog. Forsk. Sver..

[80] Matta C. (2022). Tolkningens metodologi och selektiv abduktion, The methodology of interpretation and selective abduction. Pedagog. Forsk. Sver..

[81] Alvesson M., Sköldberg K. (2008). Tolkning och Reflektion: Vetenskapsfilosofi och Kvalitativ Metod, Interpretation and Reflection.

[82] Egidius H. (2006). Termlexikon i Psykologi, Pedagogik och Psykoterapi, Glossary of Terms in Psychology, Pedagogy and Psychotherapy.

[83] Vallberg Roth A.-C., Holmberg Y., Löf C., Palla L., Stensson C. (2019). Flerstämmig Didaktisk Modellering i Förskolan, Multivocal Didactic Modelling in Preschool.

[84] Iphofen R. (2020). Handbook of Research Ethics and Scientific Integrityp.

[85] Markham A.N., Buchanan E. (2012). Ethical Decision-Making and Internet Research: Recommendations from the AoIR Ethics Working Committee (Version 2). http://aoir.org/reports/etics2.pdf.

[86] Swedish Research Council (2002). Forskningsetiska Principer Inom Humanistisk-Samhällsvetenskaplig Forskning, Principles of Research Ethics in the Humanities and Social Sciences.

[87] Swedish Research Council (2018). God Forskningssed, Good Research Practice.

[88] Ackesjö H. (2016). Övergångar Mellan Skolformer—Kontinuitet och Progression Från Förskola Till Skola, Transitions between School Forms.

[89] Comenius J.A. (1632/1989). Modersskolan Eller om Barns Omvårdnad och Fostran under de Sex Första Levnadsåren, The Mother School or about Children’s Care and Upbringing during the First Six Years of Life (Översättning och Inledning av Tomas Kroksmark).

[90] Comenius J.A. (1657/1989). Didactica Magna: Stora Undervisningsläran (Översättning och Inledning av Tomas Kroksmark).

[91] (2018). SKOLFS, Skolverkets författningssamling (2018:50). Förordning om Läroplan för Förskolan.

[92] Vallberg Roth A.-C., Åsén G. (2022). Flerstämmig kvalitet, Multivocal quality. Perspektiv på Systematiskt Kvalitetsarbete.

[93] Marton F. (2015). Necessary Conditions of Learning.

[94] Colucci-Gray L., Burnard P., Gray D., Cooke C. (2019). A Critical Review of STEAM (Science, Technology, Engineering, Arts, and Mathematics). Oxford Research Encyclopedia of Education.

[95] Palmer A. (2020). Hur Blir Man Matematisk? Att Skapa Nya Relationer till Matematik och Genus i Arbetet Med Yngre Barn, How do you become mathematical?.

[96] Imsen G. (1999). Lärarens Värld: Introduktion till Allmän Didaktik, The Teacher’s World.

[97] Bornemark J. (2020). Horisonten Finns Alltid Kvar—Om Det Bortglömda Omdömet, The horizon is Always There.

[98] Englund T. (2018). Två artskilda perioder för pedagogisk forskning: Det utbildningspolitiska systemskiftets juridiska dimension och dess djupgående konsekvenser för samhälle, skola och pedagogik, Two distinctive periods for educational research. Pedagog. Forsk. I Sver..

[99] Gustavsson B. (2002). Vad är Kunskap? En Diskussion om Praktisk och Teoretisk Kunskap, What is knowledge?.

[100] Elbow P., Weber C. (2006). The Believing Game and How to Make Conflicting Opinions More Fruitful. Nurturing the Peacemakers in Our Students: A Guide to Teaching Peace, Empathy, and Understanding.

[101] Ferraris M. (2014). Manifest för en Ny Realism, Manifest for a New Realism.

[102] Larsson S. (2009). A pluralist view of generalization in qualitative research. Int. J. Res. Methods Educ..

[103] Enthoven M., de Bruijn E. (2010). Beyond Locality: The creation of public practice based knowledge through practitioner research in professional learning communities and communities of practice: A review of three books on practitioner research and professional communities. Educ. Action Res..

